# Special Pathology of the Wisdom Teeth

**Published:** 1888-07

**Authors:** William Rushton


ARTICLE II.

### SPECIAL PATHOLOGY OF THE WISDOM TEETH.

BY WILLIAM RUSHTON.

(A Paper read before the Students' Society of the National Dental College.)

*Mr. President and Gentlemen,*—The subject of my paper tonight is the "Special Pathology of the Wisdom Teeth," and I hope you will bear with me while I briefly touch upon a few points which I hope will be of interest.

The question might naturally be asked, "Why have wisdom teeth a *special* pathology, differing from their fellows in the mouth?" I think that two reasons may be assigned; one, that the jaw, as a rule, when wisdom teeth trouble exists, is too contracted to contain its full complement of teeth, therefore the wisdom teeth, which come into place last, have more or less difficulty in eruption, and consequently cause more or less disturbance; the other reason is the close proximity of the wisdom teeth (especially the lower) to the branches of the fifth nerve. Let us inquire how it comes about that the jaw is too contracted to contain its full complement of teeth. Darwin, in his work, the "Descent of Man," gives the following reasons:—" The early male forefathers of man were probably furnished with great canine teeth, but as they gradually acquired the habit of using stones, clubs and other weapons for fighting with their enemies or rivals, they would use their jaws and teeth less and less. In this case the jaws, together with the teeth, would become reduced in size, as we may feel almost sure from innumerable analogous cases." Mr. Darwin endeavours to show that the disuse of the jaws in sexual warfare tended to their diminution in size and power. The following extract from the same work will show that partial disuse of the jaws in mastication is also an important factor. He says: "It appears as if the posterior molars or wisdom teeth were tending to become rudimentary in the more civilised races of man. I have been assured that they are much more liable to decay, and are earlier lost than the other teeth. They are also much more liable to vary, both in structure and in the period of their development, than the other teeth. Professor Schaafhausen accounts for the difference between the races by 'the posterior dental portion of the jaw being always shortened in those that are civilized,' and this shortening may, I presume, be attributed to civilized men habitually feeding on soft cooked food, and thus using the jaws less." Speaking again of the effects of the increased use and disuse of parts, the same writer says: "From the correlation which exists at least in some cases between the development of the extremities and of the jaws, it is possible that in those classes which do not labour much with their hands and feet, the jaws would be reduced in size from this cause. That they are generally smaller in refined and civilized men than in hardworking men and savages is certain." Thus, then, we have three more or less cogent reasons for the diminution in size of the jaws. Add to these the fact that in civilized races men are apt to choose wives with small jaws, that is to say, of a refined cast of feature, therefore the offspring will be sure to have this feature reproduced more or less. The jaws being of a tissue more yielding than that of the teeth, changes take place in the former more rapidly than in the dense dentinal tissues, and hence we have the phenomenon of an overcrowded dental arch

Next let us consider the effects of this overcrowding upon the wisdom teeth. First, difficult and painful eruption. The eruption of wisdom teeth is often the cause of pain and even of serious illness. Neuralgic and rheumatic pains, swelling, inflammation and even ulceration may occur. The pain is not always confined to the affected part, but may be felt in the ear and has been known to proceed as far as the clavicle, while the inflammation may affect the tonsils. Wedl speaks of difficult eruption of wisdom teeth being accompanied by a very obstinate cough like whooping cough, and of cases in which very obstinate attacks of dysentric diarrhœa which continued five, six or seven months have occurred. The eruption of the upper wisdom tooth is not as a rule so painful as that of the lower. In the upper the malposed tooth generally points backwards or outwards, and the worst that happens is the contusion of the opposing mucous membrane about the base of the coronoid process or of that of the cheek. In the lower, where it is a more serious matter, the trouble may not be caused so much by the abnormal position of the crown of the tooth (as in the upper jaw), but rather because the horizontal ramus of the jaw is so short that the crown can only be partially erupted, if at all. Besides, the ramus of the lower jaw is of dense unyielding bone, while the tuberosity of the upper is of a more cancellous structure. When the jaws are brought together the mucous membrane is contused and great pain, inflammation and suppuration may ensue, the inflammation proceeding to the adjoining tissues of the cheek and fauces. Trismus or tonic spasm of the muscles of the jaw sometimes supervenes and the lymphatic glands may become swollen and tender.

M. Nelaton and other writers quote cases in which the symptoms in painful eruption of wisdom teeth have been mistaken for scrofulous caries of the jaws, for syphilis and for cancer.

Secondly, impacted and unerupted wisdom teeth. Wisdom teeth in the lower jaw are often impacted between the dense unyielding ramus and the also unyielding second molar, giving rise to symptoms of different degrees of severity. As a rule the tooth in these cases has a foward horizontal direction, in consequence of its vertical growth being prevented by the resistance of the bone above it. It is thus tightly wedged. The consequences of this are chiefly the following: Neuralgic pains, swelling, inflammation and suppuration, trismus, dentigerous cysts; caries, absorption and necrosis of the second molar. Instances are not wanting in which partial paralysis, epilepsy, delirium, mental derangement and suicide have been the results of impaction, and I think to these results may be added the danger of the performance of severe operations, through the cause of trouble not being properly understood. I shall briefly quote a few cases in point. I have here a specimen of an impacted wisdom tooth, kindly given me by Mr. Pattinson, which was the cause of much suffering of a neuralgic kind. The wisdom tooth was buried beneath the gum, and extensively carious. Mr. Pattinson, finding that it was impossible to extract the wisdom tooth without previously extracting the second molar, did so, the result being complete relief. Mr. Salter gives an instance of suppuration extending along the jaw from an impacted wisdom tooth, accompanied by pain and swelling, with nearly complete closure of the lower jaw. The patient had been attended by a practitioner who had mistaken the cause of his trouble. His face was permanently disfigured by cicatrices, and in their midst was a mass of pouting granulations about the size of half-a-crown, from which pus was being discharged. On exploration with a probe the cause of the trouble was found deeply inpacted at the base of the coronoid process, the tooth pointing forwards and outwards. After gradually wedging the mouth open for a period of three weeks, the tooth was with great difficulty extracted through the mouth, thus saving the patient from a severe operation. Mr. Coleman mentions a somewhat similar case in which the cause had been overlooked, and the jaw had been trephined in the hope of opening up an abscess. Relief was obtained when the cause of trouble (a tooth) was removed. Trismus is not unfrequently a consequence of impacted wisdom teeth. But, first of all. I should like to remark that the definition of trismus among writers on dentistry seems to vary. Mr. Salter terms it a spasmodic contraction of the muscles of the jaw, continuous and of a truly tonic character. Mr. Heath says the term, as generally used, is etymologically incorrect, as it is applicable to the grinding and gnashing of the teeth in convulsions rather than to the tonic spasm of tetanus. Mr. Morgan Hughes relates a case occuring in the practice of Dr. Mapother, of Dublin, in which a lady of twenty-four suffered from trismus for eighteen months, due to impaction of the lower wisdom tooth (Mr. Hughes does not say whether the closure was mechanical as well as reflex). The central incisors could only be separated two lines. The second molar and wisdom tooth were extracted, and, although the trismus had lasted so long, the stiffness soon subsided. In ordinary cases of dentigerous cysts, the cause is very obscure, but, as Salter says, "When a wisdom tooth is impacted and held back behind a second molor, the irritation consequent upon a restrained position may account for it;" and there is no doubt that dentigerous cysts, resulting from impacted wisdom teeth, are comparatively frequent and serious. I quote a case taken from a memoir of Dr. Forget, of Paris, showing to what extent dentigerous cysts of this sort may go. The patient, a woman, exhibited a swelling of the right half of the lower jaw about the size and shape of a hen's egg, bounded in front by the second incisor and behind by the coronoid process. Several of her teeth on the same side had fallen out, and she had more or less pain for ten years. Lisfranc resected the half of the jaw, and the patient was discharged cured. The examination of the section showed a development of the body of the jaw, formed by the two tables of the maxilla, which were very thin and reduced at many points to the mere thickness of the periosteum, and filled with carious and purulent fluid. The bottom formed by the base of the bone, very much widened, presented in projection the crown of the wisdom tooth firmly enclosed in the base of the coronoid process. The anomalous position and regular development exhibited by the tooth in this vicious situation can leave no doubt as to the part it had played in the production and evolution of disease of the bone. It could not become enlarged without exerting a continual pressure on the neighbouriug teeth, causing prolonged suffering, inflammation of the gums, decay, loosening and spontaneous falling out of the other teeth.

Sir John Tomes and Messrs. Salter and Coleman all mention cases in which the pressure of the impacted wisdom tooth has produced absorption, erosion, or necrosis in the second molar, and they all give one the impression that this takes place through the direct pressure of the wisdom tooth. Sir John Tomes says: "The posterior fang of the second molar was much eroded by the pressure of the wisdom tooth." Mr. Coleman gives an example of an unerupted wisdom tooth, "which by pressure has caused absorption of the approximal surface of a second molar." Mr. Salter says: "The posterior fang of the second molar is apt to be eroded by absorption (caused by an impacted wisdom tooth), and the fang may be so much absorbed as to open the pulp cavity of the tooth, and even to pass through it and erode the posterior surface of the right fang." That these erosions or absorptions do take place there can be no doubt, but that they are made by the direct pressure of the wisdom tooth is open to question. I should like to hear some opinions on this point.

I have now enumerated the chief troubles arising from impacted and unerupted wisdom teeth. Such teeth very often give no troble, and they are frequently found out only by accident—as in the remarkable specimen of Sir E. Saunders, in which the wisdom teeth were found imbedded high up in the ramus, the crown reaching nearly to the level of the sigmoid notch—but as a rule they do give trouble sooner or later.

I shall now pass on to irregularities in size and shape of the wisdom teeth, including exostosis and enamel nodules, and union of second and third molars, together with special accidents in their extraction. I shall, also, briefly mention the points to be taken into consideration when deciding whether to extract the wisdom tooth or the second molar, and lastly I shall mention a case of reversion of the wisdom tooth.

We have it on the authority of Professor Flower that the wisdom tooth is, of all others, the one most frequently found suppressed. It is also the tooth in which the greatest variation takes place, both in size and shape. I cannot do better than quote the words of Sir John Tomes: "No rule can be laid down for the form and number of the roots of the *dentes sapientiæ*, so variable and inconstant are the forms assumed by these teeth. In one case the tooth is terminated by a single conical root; in another, the one is replaced by five or even six small roots." Thus we see that the variation from the typical molar found in apes and savages is very great, and there can be no doubt that in succeeding generations these teeth will be lost. When the wisdom tooth is abnormally small, it is on this account that it is tolerated in the often contracted jaw; but when on the other hand, it is normal or abnormally large, the trouble that it brings, both in and after eruption, has already been dwelt upon. Although exostosis and enamel nodules do not belong to the special pathology of the wisdom teeth strictly speaking, yet when we consider the close connection of those teeth to the branches of the fifth nerve, we can understand how they cause more intense suffering than similar disease in the first or second molars. I have here a specimen of an upper wisdom tooth, kindly placed at my disposal by Mr. Boyd Wallis, in which a small enamel nodule was the cause of many years of intense neuralgia, for which advice had been sought from the first physicians of the day. Mr. Wallis extracted the tooth, not with the idea of relieving the neuralgia, but because of the malposition and consequent worthlessness of the tooth. However, the extraction removed the cause of suffering, and the patient has been relieved ever since. Here is a tooth, a lower wisdom, which, as you will see, is much exostosed, and was the cause of closure of the jaws and much suffering. The tooth was lent to me by Mr. Marcus Davis by whom it was extracted withgreat difficulty.

The cases in which cemental union has taken place between the wisdom tooth and second molar are somewhat numerous, but sometimes the union takes place between the dentine and enamel of the respective teeth; in this case the teeth have a common pulp chamber. In the cemental union junction has no doubt taken place after the crowns were formed and while the roots were forming. In the latter case the more intimate union has taken place in the early stages of development. I have two cases here of cemental union kindly lent me by Mr. Gaddes, and I have endeavoured to depict some somewhat similar ones copied from Tomes and Wedl, one case being that of a supernumerary wisdom tooth cemented to the normal one. In these abnormally formed wisdom teeth there are always two dangers attending their extraction. In the upper jaw there is the danger of damaging the tuberosity, and in the lower the danger of injuring the inferior dental nerve. In cases like those we have just seen, great care should be exercised if extra resistance be shown when using the forceps, as the carrying away the tuberosity may result in complete deafness on the injured side. For the same reason the elevator should not be employed to an upper wisdom, though in the lower it is invaluable, as so often the backward curve of the roots allow of the tooth being displaced with comparative ease. In the lower jaw the inferior dental canal may perforate the fang of the wisdom tooth, so that an extraction may cause loss of sensation on the same side for greater or less periods. Or, in a difficult extraction of a lower wisdom tooth, the nerve trunk may be injured.

As a rule, when there is a doubt as to whether the second or third molars should be extracted, it is better that the wisdom tooth should be removed, as it is more liable to decay and is not so useful for mastication. But if, on the other hand, the second molar is carious or loose or necrosed, or it is impossible to extract the wisdom tooth through closure of the jaws or impaction, the second molar should be removed. In cases of suppuration caused by the wisdom tooth and involving the second molar, the wisdom tooth should be extracted.

I shall now speak briefly of reversion of the wisdom tooth—that is to say, of cases in which the tooth has been formed upside down. Sir John Tomes is the only author whom I have consulted who mentions the fact at all. He gives two cases, one being a reverted wisdom tooth, the fangs of which were interlocked with those of the second molar and extracted with it. The other case is one in which the crown was directed outwards towards the cheek; therefore, I should say that it was not a case in which, as he says, "the direction was completely reversed," because for *complete* reversal the crown should be buried in the jaw and the root should point towards the gum. I have here a tooth which I extracted from a patient in this hospital, a woman of fifty-one. There was a tense painless swelling on the left side of the palate, consequently the removal of several molar and bicuspid stumps on the affected side was accomplished, the result being the subsidence of the swelling. Before I extracted this tooth I naturally thought it was a *wisdom tooth stump*, instead of which I found to my surprise that the tooth had erupted fang foremost and that the crown had been buried in the alveolus from which I had dislodged it. Upon enquiry I found that the tooth had never given trouble (unless the swelling spoken of was the result of this tooth), and it had evidently taken its share in mastication, as we may judge by the way in which its fang is worn down and the high polish it has acquired. This case differs from those mentioned by Tomes in that the tooth had no connection at all with the second molar and it was not horizontal or oblique, but simply upside down, the crown where the root ought to be and *vice versa*. The crown is perfectly formed and the pulp canal is closed by formation of secondary dentine. The points I should like briefly to discuss are these: What caused the reversion of the tooth? How can we account for the eruption of the tooth in a reversed direction? When was the secondary dentine formed? What prevented the tooth being rejected as a foreign body?

I can give no satisfactory theory of its reversion, except that the wandering of the enamel germ, in the first instance, together with the pressure of the second molar, may have been the cause. As to the cause of its eruption fang first, I think that as so little is known about the forces that regulate the normal eruption of teeth, that it is almost useless to theorise about them when appearing as this has done. It may be probable that no eruption has taken place at all, but that the gum has recoiled sufficiently to lay bare the fang. The occurrence of secondary dentine, I think, is very interesting. It must have been formed prior to the eruption of the fangs, as, of course, masticating would have been fatal to the pulp, and the pulp must have been alive when it produced the secondary dentine. I can only conceive that the abnormal pressure which, I presume, altered the position of the tooth would tend to set up irritation of the surrounding parts, which irritation being transmitted to the pulp, was sufficient to cause the secondary dentine and consequently no pain on eruption.

And lastly, what prevented the tooth beidg rejected? The pulp was dead, the crown—through which no nutriment or connection could, as most authors aver, take place with the periosteum—was fairly large, and against these we have a very narrow band of living cementum. I suppose the reason is that there was just sufficient vitality remaining in this narrow strip of cementum which, taken in combination with the fact that the thick end of the wedge was locked in the alveolus instead of the thin one (as is usual), was sufficient to prevent the rejection of this remarkable tooth.

In conclusion, I must express my thanks to the many friends who have helped me by lending me specimens and in other ways, and I must also thank you all for the patience you have shown during the reading of this paper. I have no doubt there are points which I have omitted and others upon which I might have dilated more fully. The subject is a very interesting one, and I think that any remarks, whether new or old, are serviceable. It is good for us to be told what we do not know, but it is also very useful to be reminded sometimes of what we do know. *London Dental Record.*

